# Miscellany

**Published:** 1905-11

**Authors:** 


### MISCELLANY.

THE State Civil Service Commission announces that examinations will be held December 9, 1905, for several state and county positions, among which are the following: assistant chemist, cancer laboratory, Buffalo, $720; clerk and junior clerk Erie County; physician in state hospitals and institutions, $900; trained nurse, $420 to $600 and maintaenance. The last day for filing applications is December 4. Application forms and detaild information may be obtained by addressing the chief examiner of the commission at Albany.

---

EXAMINATION to secure male hospital internes for the Panama Canal. The United States Civil Service Commission announces an examination on November 29-30, 1905, at the usual places, to secure eligibles from which to make certification to fill vacancies in the position of hospital interne under the Isthmian Canal Commission on the Isthmus of Panama.

